# Atomistic Insights into the Electrochemical Oxygen Evolution Activity of Hollandite IrO_2_ Surfaces

**DOI:** 10.1002/advs.202514939

**Published:** 2025-12-29

**Authors:** Sangseob Lee, Kisung Kang, Taehun Lee, Aloysius Soon

**Affiliations:** ^1^ Department of Materials Science & Engineering Yonsei University Seoul 03722 Republic of Korea; ^2^ School of Materials Science & Engineering Chonnam National University Gwangju 61186 Republic of Korea; ^3^ Division of Advanced Materials Engineering Jeonbuk National University Jeonju 54896 Republic of Korea; ^4^ Hydrogen and Fuel Cell Research Center Jeonbuk National University Jeonbuk 54896 Republic of Korea

**Keywords:** grand‐canonical density functional theory, hollandite phase, iridium oxide, oxygen evolution reaction (OER)

## Abstract

Lowering the overpotential for the oxygen‐evolution reaction (OER) is central to designing efficient water‐splitting catalysts. However, the atomistic origin behind the enhanced OER activity of hollandite IrO_2_ compared to rutile has remained unclear. Here, using grand‐canonical DFT with an implicit solvation model, the electrochemical stability and reactivity of the most stable hollandite facets, (100) and (112) are elucidated. The thermodynamic analysis identifies that hollandite is more readily oxidized than rutile under the working potential of 1.6 V and predicts potential‐driven deintercalation of K^+^ from Hol(112) surface. Fully K‐deintercalated hollandite surfaces exhibit lower overpotentials than rutile (110) due to local lattice distortions that enhance π‐bonding with *O species. Additionally, the hollandite (112) surface possesses an exceptionally low O_2_ desorption energy of 0.45 eV (less than half that of rutile), pointing to a highly efficient O_2_‐release process. The theoretical predictions clarify the atomistic origin of the experimentally observed OER reactivity of the hollandite phase and provide deeper insight into structure–activity relationships in hollandite IrO_2_, providing rational design strategies for next‐generation OER catalysts.

## Graphical abstract

Lattice distortions at tunnel‐structured hollandite IrO_2_ surfaces reshape Ir‐O bonding: they weaken the σ‐bond contribution in the Ir‐*OH bond while enhancing the π contribution of the Ir‐*O bond. This orbital reorganization in hollandite surfaces stabilizes *O, facilitates the *OH → O step, and lowers the oxygen‐evolution reaction overpotential relative to rutile IrO_2_

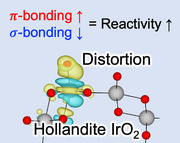

## 1 Introduction

The oxygen evolution reaction (OER) is widely recognized as the rate‐limiting step in water splitting due to its sluggish kinetics and the high overpotential required to drive it.^[^ ^1^ ^]^ Consequently, the development of efficient and stable OER electrocatalysts remains a central challenge for renewable‐energy technologies. Among transition‐metal oxides, iridium oxide (IrO_2_) has emerged as the benchmark catalyst because of its exceptional stability and high activity under acidic conditions.^[^ ^2^, ^3^, ^4^ ^]^

Although the rutile phase is the most thermodynamically stable and extensively studied structural form of IrO_2_, a variety of metastable polymorphs including perovskite, pyrochlore, columbite, and amorphous IrO_2_ phases have been explored to enhance catalytic performance by tuning the electronic structure and increasing the density of active sites.^[^ ^5^, ^6^, ^7^, ^8^ ^]^ Within this landscape, the hollandite phase stands out, characterized by 1D tunnel channels capable of hosting alkali cations such as K^+^ and Rb^+^.^[^ ^9^, ^10^, ^11^ ^]^ Notably, experimental studies report that hollandite IrO_2_ exhibits an OER overpotential approximately 40–60 mV lower than that of rutile‐type IrO_2_.^[^ ^12^, ^13^, ^14^ ^]^ In addition, hollandite‐like motifs are frequently identified in highly active amorphous iridium oxides, and their presence has been invoked to explain the exceptional catalytic activity of the amorphous phase.^[^ ^15^, ^16^, ^17^, ^18^ ^]^ Despite these observations, the atomistic origins of the activity enhancements associated with hollandite motifs remain unclear.

Another dimension of complexity in understanding this surface system arises from the intercalation chemistry of cations (i.e., K^+^) within the tunnel channels. In bulk hollandite, alkali cations modulate both structural stability and electronic properties;^[^ ^19^ ^]^ however, their influence on surface geometries, adsorbate energetics, and reaction pathways under electrochemical conditions remains poorly understood. Whereas the surface chemistry of rutile IrO_2_ is well established from both theoretical and experimental perspectives, comparable insight into hollandite surfaces is still lacking.

Modeling electrochemically active oxide surfaces under OER operational conditions poses a particular challenge: solvation, electrode potential, and field‐induced lattice distortions are all strongly coupled under operating conditions. Most prior computational studies have relied on the vacuum‐condition‐based computational hydrogen electrode (CHE) approach introduced by Nørskov et al. to estimate OER energetics.^[^ ^20^, ^21^ ^]^ While the CHE is efficient, it approximates the applied potential as a linear energy shift and neglects solvent stabilization, often missing key structural and energetic responses of complex oxide surfaces.^[^ ^22^ ^]^ Recently, to build a comprehensive multiscale model of the electrochemical interface, it has become essential to account for the electrode potential, the aqueous interface, and mass transport.^[^ ^23^, ^24^ ^]^ Among these, constant‐potential grand‐canonical (GC) DFT treats interfacial charge transfer explicitly by fixing the electron chemical potential (i.e., the electrode potential). When combined with implicit electrolyte models, it provides a more direct link to experimental observations.^[^ ^25^ ^]^ In this scheme, the electrolyte is modeled as a polarizable dielectric medium that captures solvation effects, and the electrode potential is set directly by adjusting the electron count in the grand‐canonical ensemble. As such, the GC method provides results in closer agreement with experiments and has become a standard, reliable approach for studying the Ir oxide system^[^ ^26^, ^27^, ^28^ ^]^ and achieves closer, more quantitative agreement with experiment than CHE models.^[^ ^26^, ^29^ ^]^

In this work, to model electrochemically active hollandite surfaces with and without K ions under realistic OER conditions, we employ GC‐DFT coupled with an implicit solvent. We first validate the approach by re‐examining the benchmark rutile IrO_2_(110) surface, identifying its most stable adsorbate configurations and corresponding overpotential under the OER operational conditions. We then turn to hollandite IrO_2_ surfaces, systematically evaluating how channel geometry and its K‐ion occupancy modulate the thermodynamic stability of key surface adsorbates associated with OER. Moreover, our theoretical predictions show that K‐deintercalated hollandite surfaces exhibit lower overpotentials than the rutile (110), due to local lattice distortions that enhance π‐bonding with *O species. By contrast, K‐intercalation in the ion channels raises the overpotential through destabilizing *O and altering the surface stability of *OOH. These insights provide a mechanistic understanding of hollandite's catalytic behavior and offer rational design principles for improving the activity of iridium‐based OER catalysts.

## 2 Results and Discussion

### 2.1 Surface Stability of Hollandite IrO_2_ under OER Conditions

**Figure 1:**
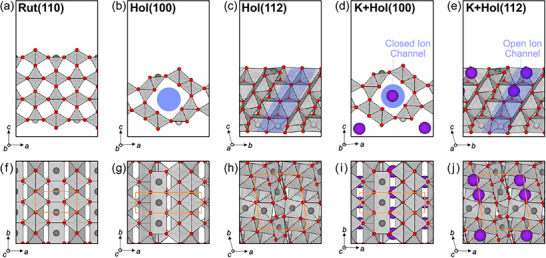
Atomic structures of rutile and hollandite IrO_2_ surfaces. Side views of a) Rut(110), b) Hol(100), c) Hol(112), d) K+Hol(100), and e) K+Hol(112); top views of f) Rut(110), g) Hol(100), h) Hol(112), i) K+Hol(100), and j) K+Hol(112). Ir, O, and K atoms are shown as gray, red, and purple spheres, respectively, and the IrO_6_ octahedra are shaded in gray. Ion channels are highlighted in blue.

We first investigated the surface free energies and relaxed geometries of five hollandite (Hol) IrO_2_ terminations: Hol(100), Hol(010), Hol(101), Hol(111), and Hol(112) (**Figure** **1**; Figure S1, Supporting Information ). Hol(100) and Hol(101) do not expose their one‐dimensional tunnels to vacuum; the ion channels (highlighted as blue in Figure 1) remain enclosed by IrO_6_ octahedra. We therefore refer to these as closed surfaces. The other three terminations (Hol(010), Hol(111), Hol(112)) allow the K atom to be extruded along the tunnel labeled as open surfaces.

Among the closed surfaces, Hol(100) is the most stable, with a surface free energy of 0.066 eVÅ^−2^, followed by Hol(101) of 0.071 eVÅ^−2^. For the open surfaces group, that of Hol(112) is the lowest with 0.086 eVÅ^−2^, whereas Hol(010) and Hol(111) have higher surface energies of 0.120 and 0.135 eVÅ^−2^, respectively. This trend is consistent with earlier observations for the crystallographically analogous MnO_2_ surfaces.^[^ ^30^ ^]^ Agreeing with our prediction, Hol(100) and Hol(112) surfaces have also been experimentally observed as dominant facets in X‐ray powder diffraction and transmission electron microscopy analyses.^[^ ^11^, ^12^, ^14^ ^]^ Based on our thermodynamic predictions, Hol(100) and Hol(112), which are the most stable closed and open surfaces, respectively, were chosen as template surfaces for further catalytic investigation. We also examined both K‐intercalated and K‐deintercalated forms of Hol(100) and Hol(112). The channel in Hol(100) is parallel to the surface plane, trapping K‐ion within the lattice (Figure 1d). In contrast, in Hol(112), the tunnel opens along the surface normal (Figure 1e), creating a pathway for K ions to leach into the electrolyte under operational electrochemical conditions.

**Figure 2:**
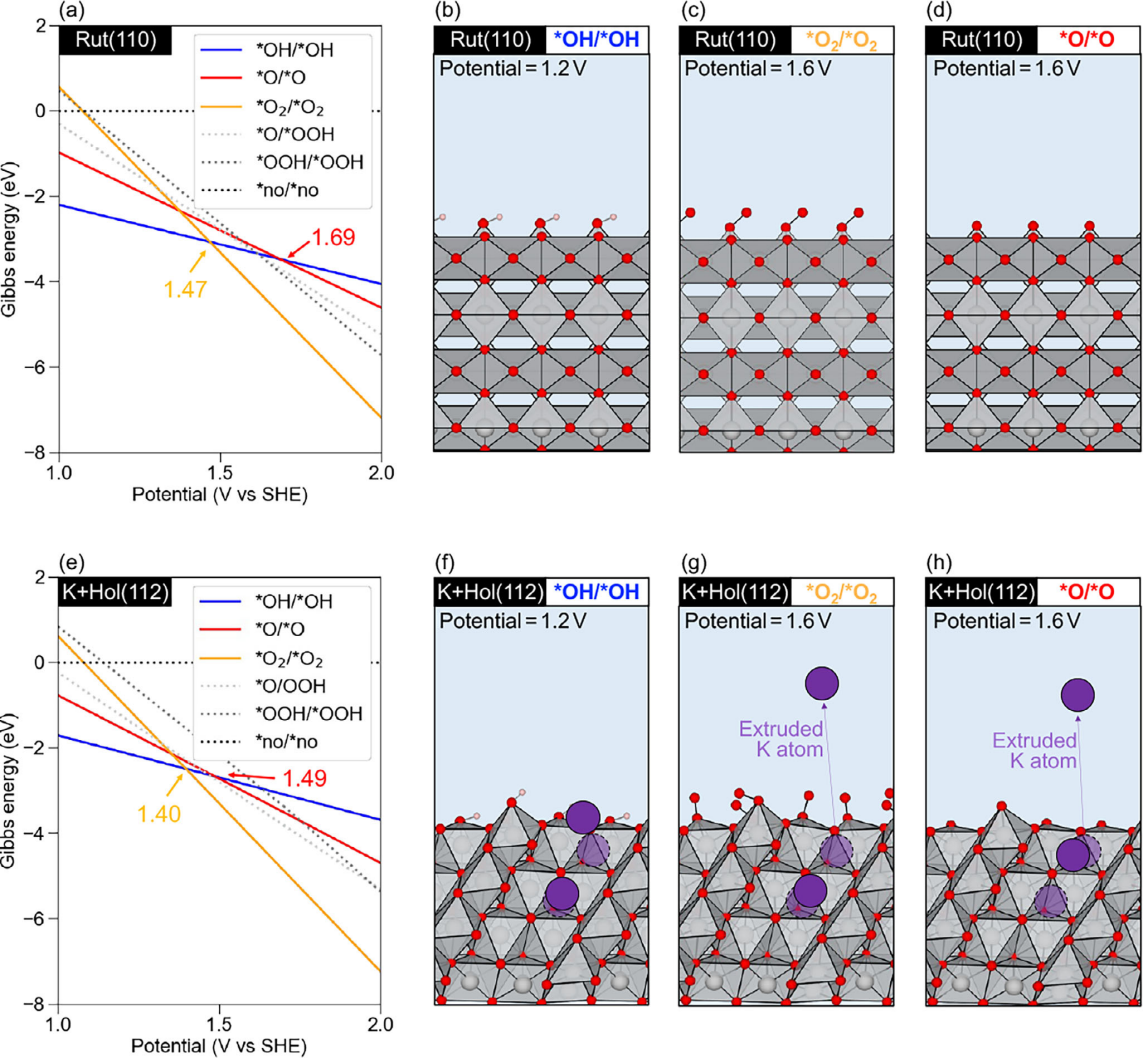
Surface Pourbaix diagrams for a) Rut(110) and e) K‐intercalated Hol(112) with (b–d) and (f–h) atomic structures of rutile and hollandite facets at various potentials (1.2 and 1.6 V). Ir, O, and K atoms are depicted as gray, red, and purple spheres, respectively. IrO_6_ octahedra are shaded in gray.

To identify the thermodynamically preferred surface adsorbates under operating electrochemical conditions, we constructed surface Pourbaix diagrams by combining an implicit solvation continuum with GC‐DFT, in which the electrode potential is set by adjusting the electron count of the slab. Adsorbates were placed on Ir coordinatively unsaturated sites (CUS) in five configurations (*OH/*OH, *O/*O, *O/*OOH, *OOH/*OOH, and *O_2_/*O_2_) representing the key OER intermediates (see Figure S2d, Supporting Information). Full details of the Pourbaix construction are provided in the Supporting Information. We first validated our GC‐DFT method on the most widely studied rutile (Rut) IrO_2_(110) surface by constructing a surface Pourbaix diagram at pH = 0 (**Figure** **2**a). This thermodynamic analysis under acidic conditions can be justified because IrO_2_ OER activity is commonly measured in acidic media.^[^ ^31^, ^32^ ^]^ Moreover, on the standard hydrogen electrode (SHE) scale, a change in pH produces only a uniform vertical shift of the Gibbs free energies, so the relative energetics among surfaces is unaffected.^[^ ^21^ ^]^

Based on previous experimental studies,^[^ ^33^, ^34^, ^35^, ^36^ ^]^ we focused particularly on ≈ 1.6 V as a representative OER operational potential. Previous CHE method studies without any consideration of the solvation effects reported that a surface fully covered by *O on Rut(110) becomes more stable than the *OH‐terminated surface at the OER‐active potentials ranging from approximately 1.6 to 2.0 V.^[^ ^26^, ^37^, ^38^, ^39^, ^40^, ^41^ ^]^ In contrast, for Rut(110), our GC‐DFT thermodynamic predictions in Figure 2a, which follow the same trend as the CHE results without a solvation model (Figure S3, Supporting Information) indicate that the *OH/*OH → *O_2_/*O_2_ transition occurs at 1.46 V (CHE) and 1.47 V (GC), whereas the subsequent *OH/*OH → *O/*O transition takes place at 1.74  and 1.69 V, respectively.

In our prediction, thermodynamically the *O_2_/*O_2_ on Rut(110) is predicted to be the most stable adsorbate configuration at the working potential of ≈1.6 V in both CHE and GC calculations (Figure 2a; Figure S3, Supporting Information), in agreement with earlier CHE + implicit solvation model calculations.^[^ ^42^ ^]^ Our theoretical predictions deviate slightly from the recent experiment. In situ XPS and resonant photoelectron spectroscopy have detected an *O/*O‐covered Rut(110) surface with minor *OH contributions, whereas molecularly adsorbed O_2_ (i.e., *O_2_/*O_2_ surface) was not observed under comparable operational conditions.^[^ ^43^, ^44^, ^45^, ^46^ ^]^ The discrepancy likely arises because the GC surface Pourbaix diagram captures only thermodynamic contribution; kinetic barriers and instrumental detection limits can prevent attainment or detection of the *O_2_/*O_2_ state.^[^ ^46^, ^47^ ^]^ Furthermore, this discrepancy may also arise from our pristine defect‐free slab model at single crystalline facet, whereas defects and surface roughness on nanoparticles can strongly affect stability of surface adsorbates.^[^ ^46^, ^48^, ^49^ ^]^

We now turn to the thermodynamically preferred adsorbates on hollandite surfaces upon applied potential. GC‐DFT surface Pourbaix diagrams of K‐intercalated and deintercalated Hol(112) and Hol(100) are plotted in Figure 2e and Figure S2 (Supporting Information). We observe that both Hol(112) and Hol(100) oxidize more readily than Rut(110): the transition potentials for Hol(112) and Hol(100) are lower than those of the rutile surface. In particular, the *OH/*OH → *O/*O crossover on K+Hol(112) occurs 0.20 V below the corresponding value for Rut(110), while the *OH/*OH → *O_2_/*O_2_ crossover is reduced by 0.07 V (cf. Figure 2a,e). Similar trends are found for Hol(100) and K+Hol(100) (see Figure S2, Supporting Information). Moreover, both hollandite terminations with and without K‐ions are dominated by a *O_2_/*O_2_‐covered surface at the working electrode potential of 1.6 V versus SHE, showing the similar behavior of Rut(110) (Figure 2e; Figure S2, Supporting Information).

Interestingly, we found that the GC implicit solvent treatment is crucial for predicting the geometry of K‐intercalated surfaces. Whereas the obtained geometry in the vacuum retains the K‐ion in the tunnel, the increase in the potential to ≈ 1.2 V drives the outermost K‐ion out of the Hol(112) channel (see Figure 2f). At ≈ 1.6 V, the surface K^+^ deintercalates and migrates from the lattice channel into the bulk water layer during geometry optimization with significant energetic gain (Figure 2g,h; Figures S4d and S5, Supporting Information). This behavior appears only on the open Hol(112) facet; the closed Hol(100) surface keeps its K‐ions within the lattice (Figure S4, Supporting Information). Moreover, Bader charge analysis confirms that the de‐intercalated K ion in the bulk water is a solvated K^+^. This finding is consistent with our earlier bulk Pourbaix prediction, which showed that deintercalated hollandite becomes thermodynamically favorable over K‐intercalated hollandite at potentials below 1 V.^[^ ^40^ ^]^ Altogether, our results indicate a strong thermodynamic driving force for near‐surface K^+^ deintercalation under the OER operating conditions.

### 2.2 OER Catalytic Activity of Hollandite IrO_2_ at Operating Conditions

In the adsorbate evolution mechanism (AEM) for OER, the Gibbs free energy can be evaluated through the reaction steps given in Equations (1)–(4):

(1)

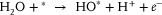

(2) $${\mathrm{HO}}^{*}\;\to \;O^{*}+H^{+}+e^{-}$$

(3) $$O^{*}+H_{2}O\;\to \;{\mathrm{HOO}}^{*}+H^{+}+e^{-}$$

(4)

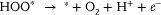




Equations (1)–(3) are proton‐coupled electron‐transfer (PCET) steps, whereas Equation (4) involves both a PCET and a chemical (CE) step. Following recent studies,^[^ ^26^, ^50^ ^]^ we separated Equation (4) into two elementary reactions:

(5) $${\mathrm{HOO}}^{*}\;\to \;O_{2}^{*}+H^{+}+e^{-}$$

(6)

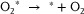

where Equation (5) is a PCET step and Equation (6) is a purely CE step. This separation allows a more precise identification of the potential‐determining step (PDS) by distinguishing between electrochemical and purely chemical steps. While alternative reaction pathways such as the lattice oxygen mechanism (LOM) are of significant interest in IrO_2_ catalysis, we focus on the adsorbate evolution mechanism, as prior work indicates that LOM is thermodynamically unfavorable on the defect‐free surfaces considered here.^[^ ^40^, ^51^ ^]^

We first examined the AEM reaction energies at zero potential within the CHE method (no solvation effect) by explicitly separating the PCET and CE steps for Rut(110), Hol(100), and Hol(112) (Figure S6, Supporting Information). For IrO_2_ Rut(110), most AEM studies evaluate the reaction free energies without separating the PCET and CE steps, and the resulting PDS has been proposed as the *OOH → * + O_2_ step. As discussed in recent work,^[^ ^28^ ^]^ the predicted PDS depends strongly on both the computational method and the starting surface structure (e.g., O‐covered versus hydroxylated). Most theoretical studies identify *OOH → * + O_2_ as the PDS for rutile IrO_2_(110).^[^ ^40^, ^51^, ^52^, ^53^, ^54^, ^55^ ^]^ When these steps are treated separately, however, the PDS of most facets shifts from *OOH → * + O_2_ to the *OH → *O oxidation (red circles in Figure S6, Supporting Information), thereby lowering the calculated overpotential (η) to 0.39–0.50 eV.

**Figure 3:**
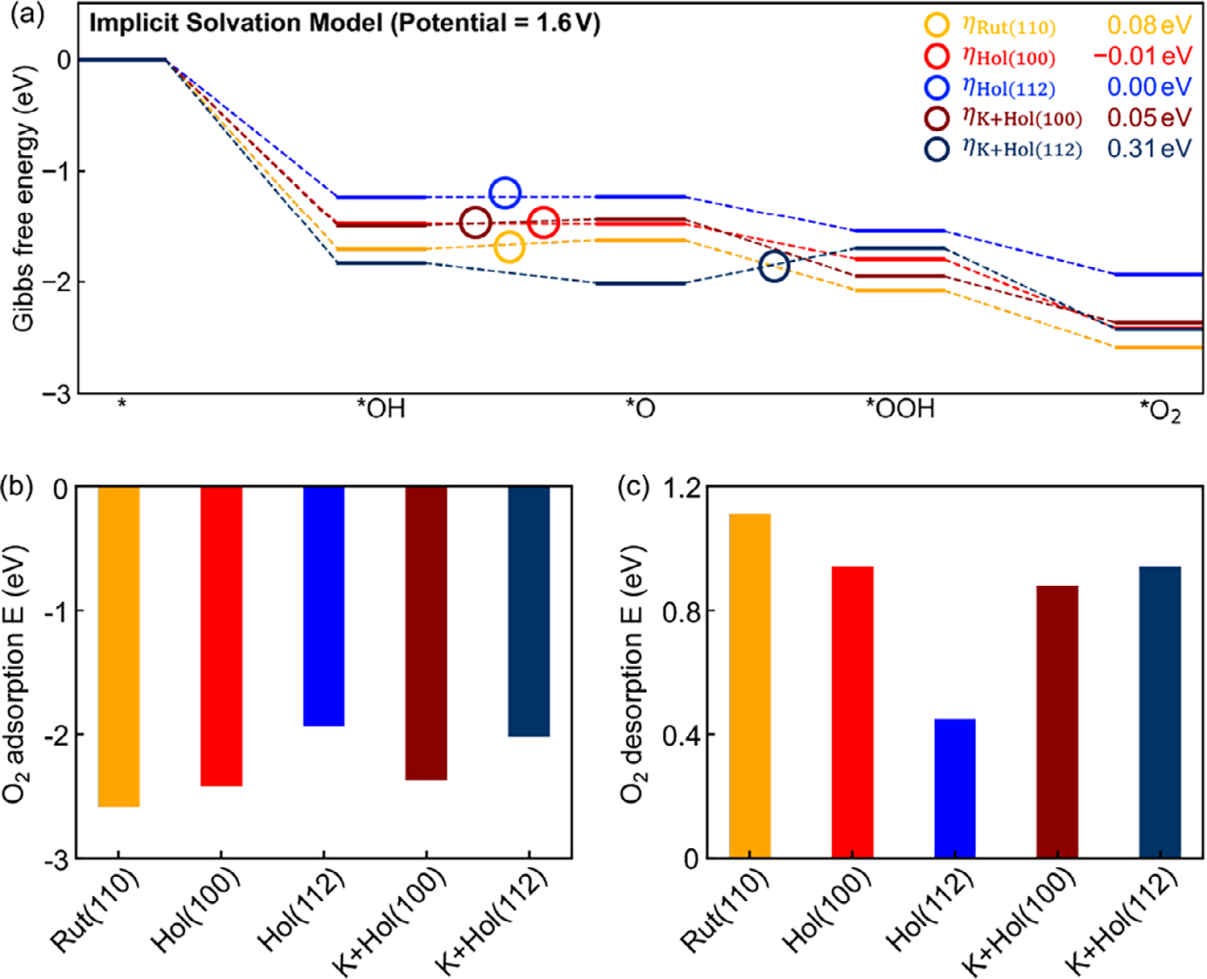
a) Gibbs free energy profile for AEM at 1.6 V, b) O_2_ adsorption energy *E*_ads_, and c) O_2_ desorption energy *E*_des_ at 1.6 V with implicit solvation. The energy barrier of the potential‐determining step is indicated by circles.

**Figure** **3**a displays the GC‐AEM reaction energies with implicit solvation model at the experimental working condition 1.6 V for Rut(110), Hol(100), K+Hol(100), Hol(112), and K+Hol(112). The diagram includes only the PCET steps in Equations (1)–(3) and (5). Under these conditions (1.6 V), Hol(100) and Hol(112) yield thermodynamically favorable OER pathways: every step is downhill (or flat), giving an overpotential of η = 0.00, −0.01 eV. By contrast, Rut(110) retains an uphill barrier, with η = 0.08 eV at 1.6 V. The result highlights the intrinsically lower η of hollandite‐type IrO_2_ relative to the rutile benchmark. Our calculated η difference between rutile and hollandite of approximately 0.08 V is in good agreement with the experimentally reported range of 0.04–0.06 V.^[^ ^12^, ^13^, ^14^ ^]^ In contrast, we found that K‐intercalation raises the overpotential with η increasing to 0.05 eV for K+Hol(100) and 0.31 eV for K+Hol(112), reducing the OER reactivity.

Except for K+Hol(112) termination, the step from *OH to *O was identified as the PDS (see circles in Figure 3a). For K+Hol(112), *O is stabilized compared to Hol(112), *OOH becomes relatively less stable, shifting the PDS to the *O → *OOH step, thereby lowering the overall reactivity. In a comparison between Hol(100) and K+Hol(100), the intercalation of K leads to a relative destabilization of *O, resulting in increased η by 0.06 eV.

We now focus on the CE step (i.e., molecular O_2_ desorption) proposed as a key bottleneck in the OER mechanism.^[^ ^56^, ^57^ ^]^ Figure 3b,c compares the O_2_ adsorption (*E*_ads_) and desorption (*E*_des_) energies for the five different surfaces. Among considered surfaces, Rut(110) shows the strongest binding, with −2.59 and 1.11 eV of *E*_ads_ and *E*_des_.

All hollandite terminations bind O_2_ more weakly than Rut(110), exhibiting less exothermic adsorption and lower *E*_des_. In particular, Hol(112) showed the lowest *E*_des_ of 0.45 eV. To assess kinetics of the O_2_ desorption process, we calculated the O_2_ desorption kinetic barrier using nudged‐elastic‐band (NEB) calculation (Figure S7, Supporting Information) and found a negligible activation energy, indicating that *E*_des_ is the descriptor for determining the O_2_ desorption reactivity.

Altogether, our GC‐DFT OER reactivity predictions indicate that Hol(100) and Hol(112) are more active OER catalytic systems than Rut(110), in line with the experimental observation.^[^ ^12^, ^13^, ^14^ ^]^ In particular, Hol(112) exhibits downhill reaction energies at 1.6 V and possesses an exceptionally low *E*_des_ of 0.45 eV, pointing to a highly efficient O_2_‐release process.

It is interesting to note the solvent effect on the AEM reaction energies. Table S1 reports the stepwise Gibbs energies at 1.6 V obtained with the vacuum CHE method and with the GC approach and the implicit solvation model. For Rut(110), Hol(100), Hol(112), K+Hol(100), and K+Hol(112), the largest solvent‐induced energy shifts are the *OOH‐related processes: the formation of *OOH (Equation 3) and the oxidation of *OOH (Equation 5). Because the *OOH intermediate is highly polar, the implicit solvent model strongly stabilizes it relative to other surface species. In our calculations, this lowers the *OOH formation free energy (Equation 3) by up to −0.33 eV and, conversely, raises the subsequent *OOH oxidation free energy (Equation 5) by up to +0.43 eV. Among the considered facets, K+Hol(112) exhibits the most significant solvent‐induced energy shifts (larger than 1.0 eV), attributed to both geometric rearrangement and the K‐deintercalation under solvated conditions. In contrast, energetics in Hol(112) is insensitive to solvation, with all stepwise differences remaining below 0.05 eV.

### 2.3 Electronic and Geometric Origins of the Catalytic Activity of Hollandite Surfaces

**Figure 4:**
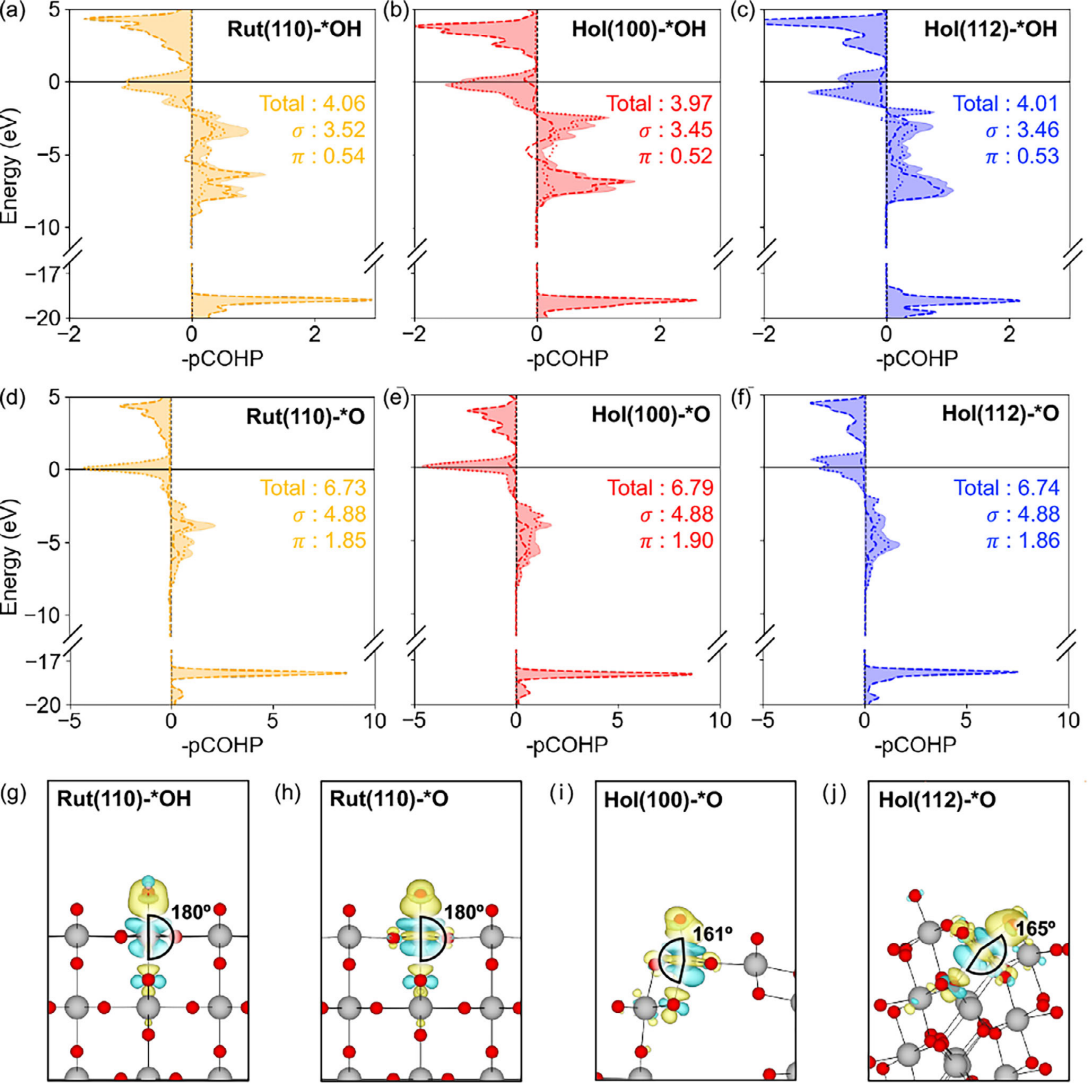
Ir–O(H) bonding analysis based on −pCOHP for a) Rut(110), b) Hol(100), and c) Hol(112) under *OH adsorption, and for d) Rut(110), e) Hol(100), and f) Hol(112) under *O adsorption. Charge density difference plots for g) *OH‐Rut(110), h) *O‐Rut(110), i) *O‐ Hol(100), and j) *O‐Hol(112). In −pCOHP plot, total −pCOHP, σ‐, and π‐contributions are shown as shaded, dashed, and dotted lines, respectively. In Charge density difference plots, yellow and cyan regions represent charge accumulation and depletion (isosurface level: ±0.005 *e*/Bohr^3^). Ir and O atoms are depicted as gray and red spheres.

To rationalize the lower η of hollandite facets, we analyzed the bonding character of the adsorbates (*OH and *O) involved in the PDS (*OH → *O) on Hol(100) and Hol(112) and compared them with Rut(110). **Figure** **4**a–f display the negative projected crystal‐orbital Hamilton population (−pCOHP) for the surface Ir–O and Ir–OH bonds; positive values indicate bonding states, whereas negative values denote antibonding states.^[^ ^58^, ^59^, ^60^ ^]^

Integrated −pCOHP values (−IpCOHP) for the Ir–O and Ir–OH bonds quantify a total bonding strength and its σ‐ and π‐bond contributions. The −IpCOHP values for considered surfaces is listed in Table S2 and S3 (Supporting Information). For *OH adsorption, the Ir_CUS_–OH σ‐bond is strongest on Rut(110), with −IpCOHP values of 3.52 for Rut(110), 3.46 and 3.45 for Hol(112) and Hol(100) (Figure 4a–c), while the π‐contribution varies only marginally. We also analyzed the Ir–O bond characteristics of the *OOH adsorbate, which follows the same trend as *OH (see Table S4, Supporting Information).

The opposite trend is observed for *O adsorption. For *O adsorption, the surface IrO_6_ octahedra on the hollandite facets stabilize the terminal O through enhanced π‐bond hybridization with the Ir_CUS_ atom. The −IpCOHP of σ‐bond contribution (4.88) of Ir_CUS_‐*O is nearly identical for Rut(110), Hol(100), and Hol(112); by contrast, the π contribution is slightly larger on the hollandite surfaces as 1.86 for Hol(112) and 1.90 for Hol(100) versus 1.85 for Rut(110) (Figure 4d–f). Therefore, hollandite facets stabilize *O via stronger π‐bonding but destabilize *OH through weaker σ‐interactions, making the *OH → *O step thermodynamically easier and lowering the overpotential of PDS. Interestingly, we observe an approximately uniform negative shift in the total −ICOHP under GC conditions at 1.6 V relative to vacuum with zero potential (see Tables S5 and S6, Supporting Information), while the relative ordering and bonding features are preserved, indicating that the enhanced Ir–O π‐bonding is intrinsic to the hollandite (channeled) structural network.

We also performed a Bader charge analysis of the surface Ir site for each surface with adsorbates (see Table S7, Supporting Information). We find an adsorbate dependent trend in the surface Ir charge state. Ir is least positively charged on the bare surface (*), most positively charged for *O (consistent with stronger Ir–O bonding), and changes for *OH/*OOH are comparatively small. Within the same facet, K intercalation generally reduces the positive Bader charge on surface Ir, with lattice K acting as an electron donor, which indicates weaker electron depletion and lower Ir–O bond ionicity.

Geometric and electronic analyses highlight how local lattice distortions govern adsorbate bonding on the two IrO_2_ polymorphs. For both rutile and hollandite surfaces, deprotonation of *OH strengthens the O–Ir_CUS_ bond by enhancing π‐hybridization. Notably, the resulting *O adsorbate induces a more pronounced lateral charge redistribution in the IrO_6_ octahedron than does *OH, reflecting stronger π‐orbital overlap (Figure 4g,h; Figure S8, Supporting Information).

However, the strength of the π‐orbital overlap introduced by *O depends sensitively on the local geometry. On Rut(110), the surface IrO_6_ octahedron retains an axial O–Ir–O angle of 180 ° for both *OH and *O, so the additional π interaction remains limited. In contrast, the surface IrO_6_ octahedra in hollandite are inherently more distorted, and this distortion increases upon deprotonation: Hol(100) and Hol(112) display bent axial angles of 165 ° and 169 ° for *OH, compared with more distorted values of 161 ° and 165 °, for *O. This increased off‐center displacement of the Ir atom in the *O state enhances lateral orbital overlap, thereby strengthening π‐bonding within the surface IrO_6_ as evidenced by the in‐plane charge delocalization in Figure 4i,j and stabilizing *O on the hollandite facets. These results indicate that local lattice distortion can be a key descriptor for predicting catalytic reactivity quantified by intra‐octahedral distortion indexes of IrO_6_ (such as, bond‐angle variance, bond‐length distortion, and quadratic elongation).^[^ ^61^ ^]^ Engineering these distortions provides a practical strategy for designing highly active catalytic surfaces.

Because we do not explicitly model the interfacial water layer at the K‐intercalated hollandite IrO_2_ surface, the hydration structure, aqueous interfacial geometry, and interfacial charge redistribution, which can influence the OER pathway and kinetics, are not resolved. In this context, an explicit‐solvent treatment of this interface is a promising direction for future work.^[^ ^62^, ^63^ ^]^

## 3 Conclusion

In conclusion, we employed grand‐canonical (GC) DFT with an implicit solvent model to elucidate the OER reactivity of hollandite IrO_2_ facets in close comparison with the rutile (110) surface. After identifying Hol(100) and Hol(112) as the most stable terminations, our surface Pourbaix diagrams revealed that at OER operational condition (1.6 V) both facets oxidize more readily than Rut(110) and the Hol(112) surface undergoes potential‐driven K^+^ deintercalation. At 1.6 V, GC‐OER reaction profiles showed that Hol(100) and especially Hol(112) exhibit lower overpotentials than rutile (110), a finding that matches recent experimental observation. Our further analysis shows that this enhanced OER reactivity on hollandite surfaces is attributed to the selective stabilization of *O over *OH, which eliminates the energy barrier for the potential‐determining step and an exceptionally low O_2_ desorption energy of Hol(112). Moreover, under applied potential in the presence of solvent, K‐ion in hollandite thermodynamically deintercalates from the channels and prefers to migrate into the water layers. Residual K‐ion trapped in the tunnels acts to reduce the OER reactivity. By addressing the challenge of modeling solvation, electrode potential, and field‐induced lattice distortions on tunnel‐structured Ir oxide hollandite surfaces, our GC‐DFT calculations explain their enhanced OER reactivity over rutile, establishing design principles for next‐generation tunnel‐structured OER catalysts.

## Conflict of Interest

The authors declare no conflict of interest.

## Supporting information

Supporting Information

## Data Availability

The data that support the findings of this study are available from the corresponding author upon reasonable request.
