# Supplementary material for: Atomistic Insights into the Electrochemical Oxygen Evolution Activity of Hollandite IrO2 Surfaces
Source: Adv Sci (Weinh). 2025 Dec 29;13(6):e14939. doi: 10.1002/advs.202514939 (PMC12866700; doi:10.1002/advs.202514939)
Supplement: Supplementary file 1 — Supporting Information [file ADVS-13-e14939-s001.pdf]

Supplementary Information

Atomistic Insights into the Electrochemical Oxygen Evolution Activity of Hollandite IrO_2 Surfaces

Sangseob Lee and Aloysius Soon*

Department of Materials Science & Engineering,

Yonsei University, Seoul 03722, Republic of Korea

Kisung Kang

School of Materials Science & Engineering,

Chonnam National University, Gwangju 61186, Republic of Korea

Taehun Lee†

Division of Advanced Materials Engineering,

Jeonbuk National University, Jeonju 54896, Republic of Korea and

Hydrogen and Fuel Cell Research Center,

Jeonbuk National University, Jeonbuk 54896, Republic of Korea

* aloysius.soon@yonsei.ac.kr

† taehun.lee@jbnu.ac.kr

METHODS

Computational Details. The DFT calculations were performed using the projector augmented wave (PAW) method [1] as implemented in the Vienna *Ab initio* Simulation Package (VASP) [2, 3]. The generalized gradient approximation (GGA) with the Perdew–Burke–Ernzerhof (PBE) exchange-correlation (xc) functional was used for the calculations, along with the DFT-D3(BJ) dispersion correction, to account for van der Waals (vdW) interactions [4, 5]. s_6, s_8, a_1, and a_2 were set to 1.0000, 0.7875, 0.4289, and 4.4407, respectively, as damping parameters. We used the VASP PAW–PBE pseudopotential (potpaw-PBE.54). A plane-wave kinetic energy cutoff of 500 eV and a Γ-centered k-point grid with a spacing of 0.15 Å^{-1} were used. The energy and force convergence criteria were set to less than 10^{-5} eV and 0.02 eV/Å, respectively.

The optimized bulk structural parameters of rutile IrO_2, $a = b = 4.51$ Å, $c = 3.18$ Å (space group $P4_2/mnm$); hollandite (Hol) IrO_2, $a = 10.23$ Å, $b = 3.18$ Å, $c = 9.98$ Å, $\beta = 90.70°$ ($C2/m$); and K-intercalated hollandite (K+Hol) IrO_2, $a = 10.03$ Å, $b = 3.20$ Å, $c = 9.89$ Å, $\beta = 90.68°$ ($C2/m$). Constructed surface models consist of at least four IrO_6 layers; the bottom two layers were fixed to bulk geometries, and a 15 Å vacuum was added along the surface normal to avoid image interactions. All structures and charge density were visualized using VESTA software [6].

To incorporate solvation and electrochemical effects in the slab models, the implicit solvation model based on the nonlinear Poisson–Boltzmann equation was used as implemented in VASPsol++ [7]. The model includes the influence of electrode potential through adjustments in the Fermi energy and the number of electrons. GC-DFT treats the system as an open ensemble in equilibrium with an electron reservoir at a fixed potential. This allows the total number of electrons in the simulation to vary, providing a much more physical representation of electron exchange under potentiostatic control and enabling the explicit modeling of charge redistribution on the electrode surface. We set solvent dielectric constant, electrolyte concentration, effective surface tension, and ionic radius as 78.4, 1 mol/L, 0 eV/Å^2, and 4 Å, respectively. The standard hydrogen electrode (SHE) reference potential was taken as −4.44 eV following previous studies [8, 9].

To calculate O_2 desorption energy barriers, we used the climbing-image nudged-elastic-band (CI-NEB) method [10]. We conducted NEB calculations at constant-potential con-

ditions (1.6 V). Since O_2 has triplet states in the gas phase, we conducted spin-polarized calculations.

The projected crystal orbital Hamilton population (pCOHP) analysis was carried out using the LOBSTER (v5.1.1) software to evaluate bonding characters [11–16]. The pCOHP is calculated as the product of the projected density matrix and the projected Hamiltonian matrix, providing insight into the bonding and antibonding character and bonding strength between atoms following below equations,

$$\mathrm{pCOHP}_{\mu v}(E, \mathbf{k}) = \sum \mathcal{R}\left[P_{\mu v j}^{(\mathrm{proj})}(\mathbf{k}) H_{v\mu}^{(\mathrm{proj})}(\mathbf{k})\right] \times \delta\left(\epsilon_j(\mathbf{k}) - E\right) \quad , \tag{1}$$

where $P_{\mu v j}^{(\mathrm{proj})}(\mathbf{k})$ and $H_{v\mu}^{(\mathrm{proj})}(\mathbf{k})$ are projected density matrix and projected Hamiltonian matrix elements for every band j and every $\mathbf{k}$-point, respectively.

Charge density difference (CDD) analysis was performed to visualize the charge redistribution upon adsorbate binding, using the equation:

$$\rho_{\mathrm{CDD}} = \rho_{\mathrm{total}} - \rho_{\mathrm{slab}} - \rho_{\mathrm{adsorbate}} \quad , \tag{2}$$

where ρ_{total}, ρ_{slab}, and $\rho_{\mathrm{adsorbate}}$ represent the charge densities of the adsorbate–slab system, the clean slab, and the isolated adsorbate, respectively.

Construction of Surface Pourbaix Diagram. To construct the Pourbaix diagram within a solvation model and grand canonical ensemble framework, we calculated various surface and adsorbate configurations under a range of applied potentials (0.8, 1.2, 1.6, 2.0, and 2.4 V). The computed energies were then fitted to quadratic equations to express the energy as a function of the electrode potential [17], as shown in Fig. S9**a** and Equation 3,

$$\Omega(\Phi) = a(\Phi)^2 + b(\Phi) + c \quad , \tag{3}$$

where Ω and Φ are the grand canonical energy and the electrode potential, respectively. Based on these fitted equations, the relative energies were evaluated with respect to the clean surface configuration without any adsorbates (*no/*no). However, the K+Hol(112) system presented a more complex behavior, as the K atom was de-intercalated into the electrolyte region at certain potentials. After de-intercalation, the system no longer followed

a simple quadratic trend. Therefore, we divided the potential range into three regions: before K extrusion, during the transition, and after extrusion. For the regions before and after extrusion, we employed separate quadratic fits, while the intermediate region was described using a linear function. Using these fitted equations, relative energies were again referenced to the clean surface configuration as shown in Fig. S8**b**.

Using GC-DFT energies, the Gibbs energy for each step of the oxygen evolution reaction (OER) was calculated using the CHE method [18, 19] with the following expression,

$$\Delta G = \Delta E + \Delta \mathrm{ZPE} + \int C_p dT - T\Delta S - eU \quad , \tag{4}$$

where ΔE is the reaction energy obtained from DFT, ΔZPE is the zero-point energy (ZPE) correction, C_p is the heat capacity of the gas-phase species, T is the temperature, ΔS is the entropy change, and U is the electrode potential.

FIGURES

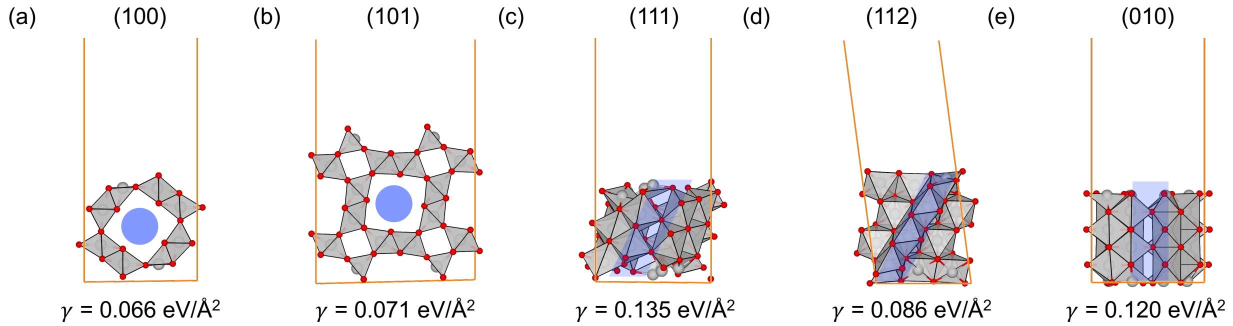

Fig S1. Atomic structure of hollandite surfaces and their surface energy for the **a** Hol(100), **b** Hol(101), **c** Hol(111), **d** Hol(112), and **e** Hol(010). The iridium, oxygen, and potassium atoms are depicted as gray and red spheres, respectively, while the IrO_6 octahedra are shaded in gray. The ion channels are shaded in blue. The unit cell is represented by the orange lines.

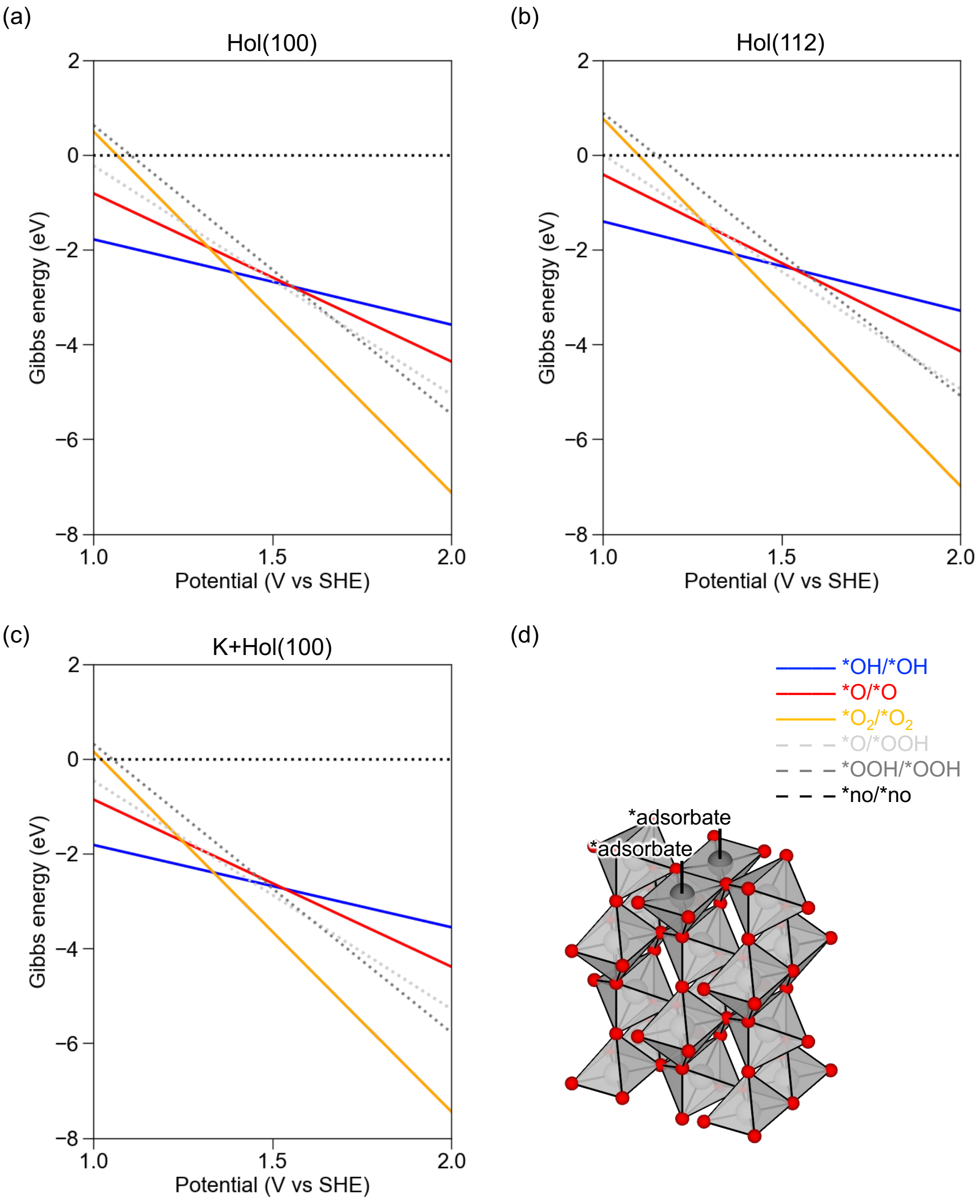

Fig S2. Surface Pourbaix diagrams obtained using the implicit solvation model and grand canonical ensemble are shown for **a** Hol(100), **b** Hol(112), and **c** K+Hol(100). **d** Schematic illustration for the considered adsorbate sites. The iridium, oxygen, and potassium atoms are depicted as yellow, red, and purple spheres, respectively, while the IrO_6 octahedra are shaded in gray.

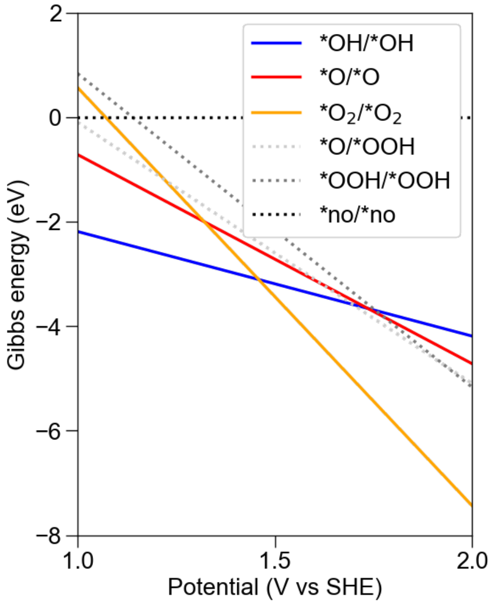

Fig S3. Surface Pourbaix diagram of Rut(110) obtained under vacuum condition.

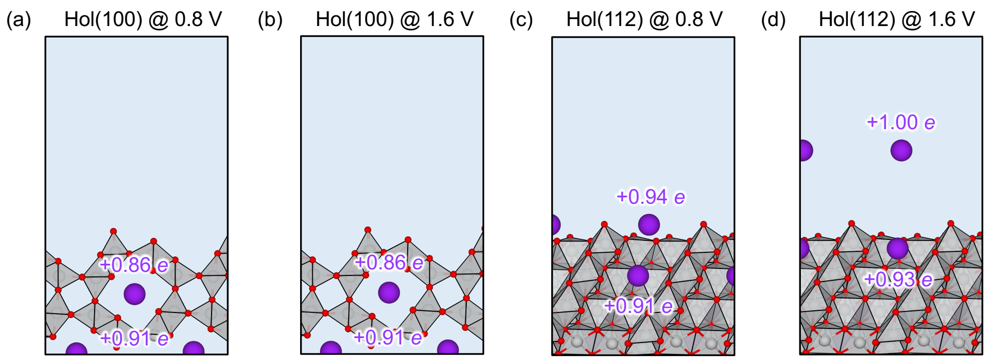

Fig S4. Atomic structures at selected potentials are shown for **a** Hol(100)-*O/*O at 0.8 V, **b** Hol(100)-*O/*O at 1.6 V, **c** Hol(112)-*O/*O at 0.8 V and **d** Hol(112)-*O/*O at 1.6 V. The iridium, oxygen, and potassium atoms are depicted as gray, red, and purple spheres, respectively, while the IrO_6 octahedra are shaded in gray. The corresponding Bader charges of potassium are indicated within each panel.

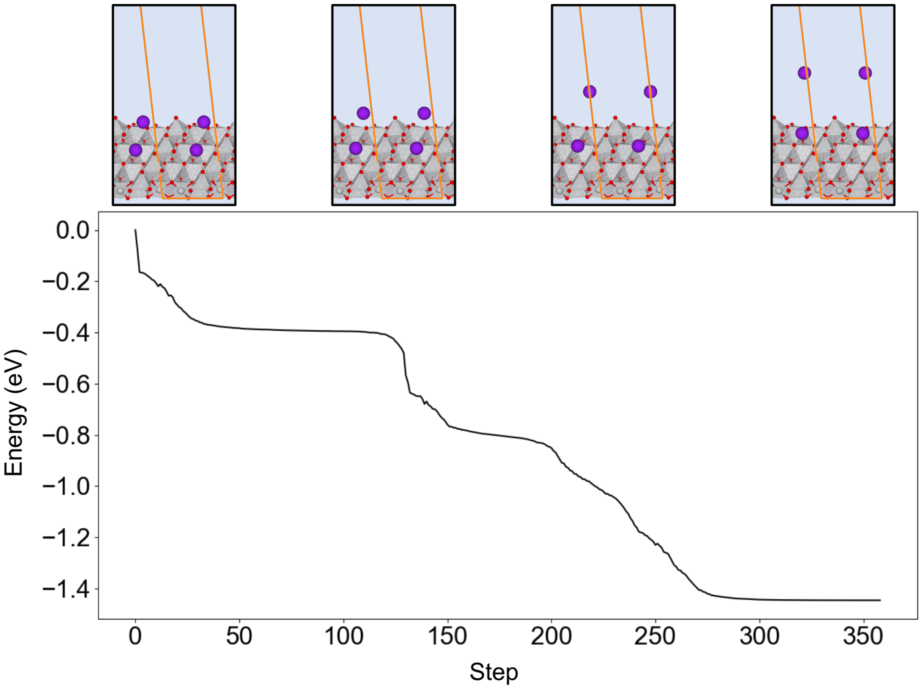

Fig S5. Snapshots of K^+ deintercalation at 1.6 V. Constant-potential GC-DFT with implicit solvent drives K^+ from the Hol(112) tunnel into the aqueous layer during geometry relaxation. Ir and O atoms are shown as gray and red spheres and IrO_6 octahedra are shaded gray. The unit cell is outlined in orange.

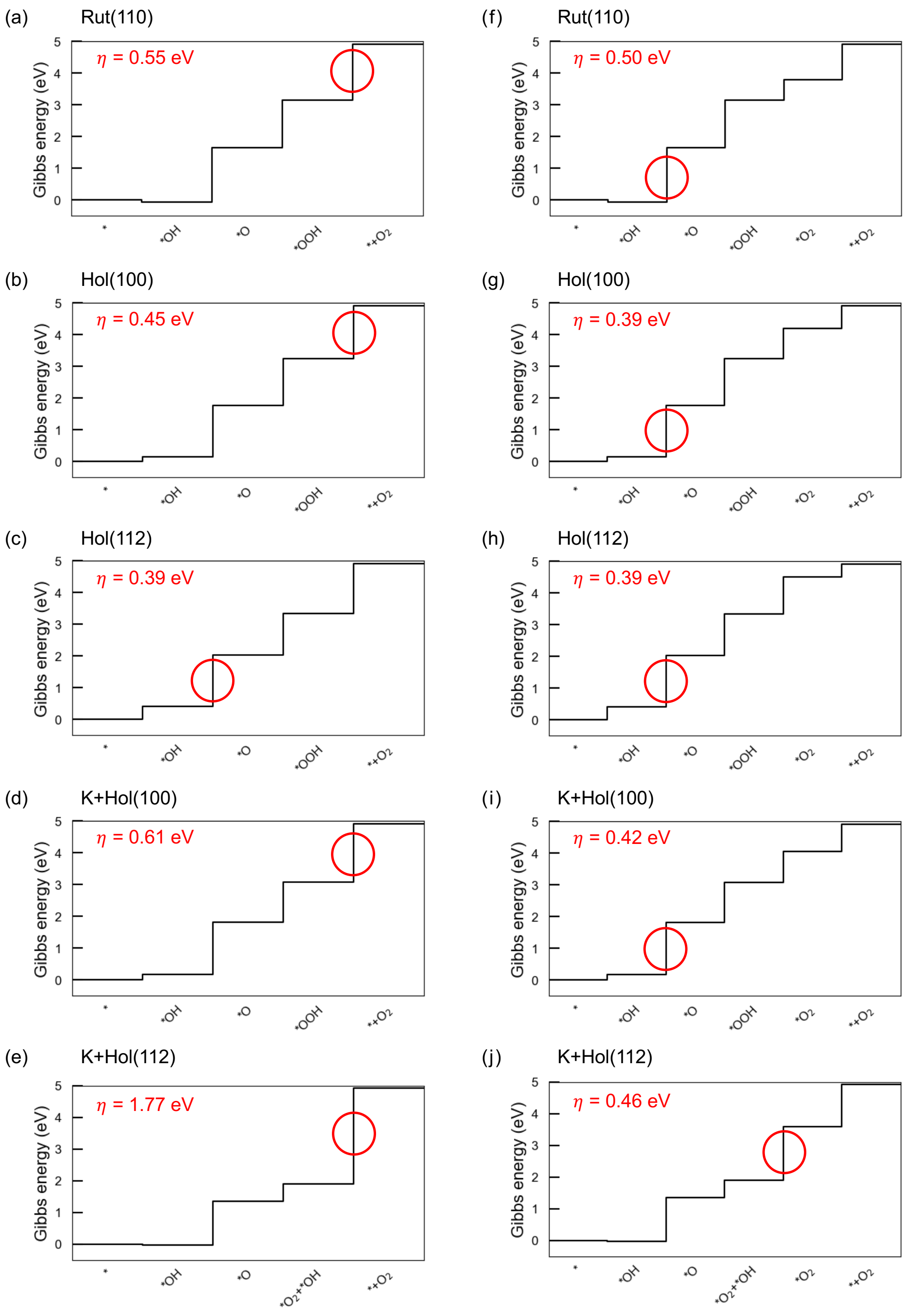

Fig S6. Gibbs energy plots of the OER without the solvation model. Panels (**a**–**e**) present results based on Equation 4 in the main text, while panels (**f**–**j**) correspond to results based on Equations 5 and 6. Potential-determining steps are highlighted with red circles, and the corresponding overpotentials (η) are indicated in red at the top left of each panel.

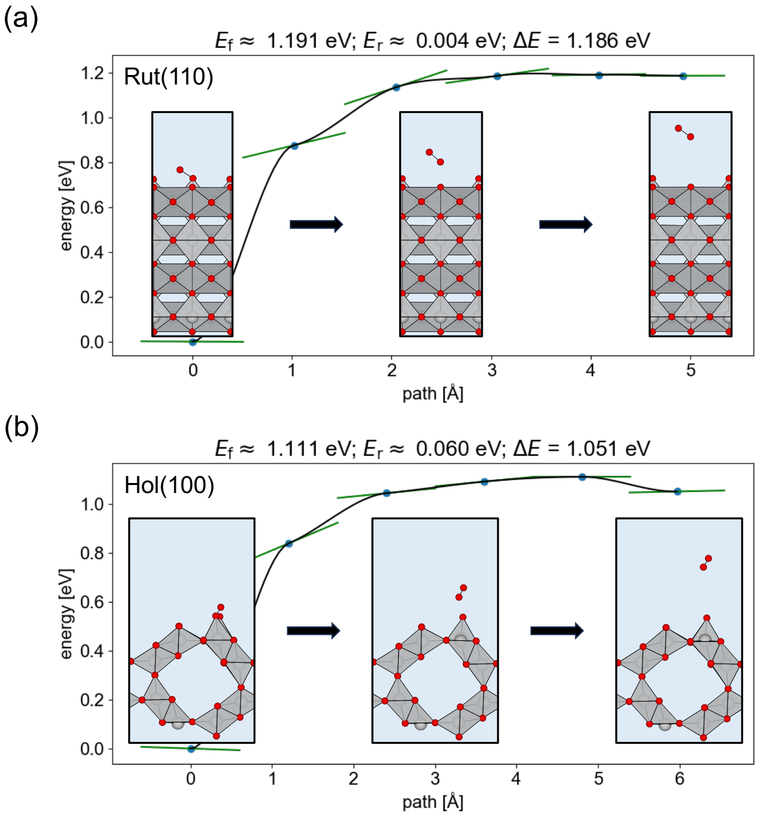

Fig S7. NEB plot of O_2 desorption for Rut(110) and Hol(100). The forward energy barrier (E_f), reverse energy barrier (E_r), and reaction energy (ΔE) are indicated at the top of each plot. The iridium and oxygen atoms are depicted as gray and red spheres, respectively, while the IrO_6 octahedra are shaded in gray.

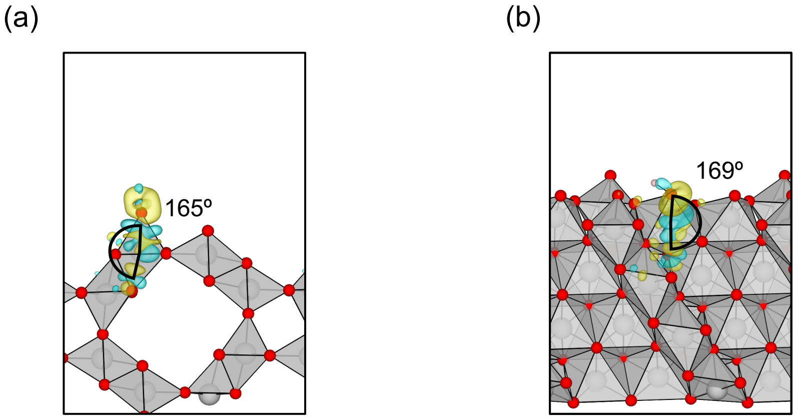

Fig S8. Charge density plots for **a** Hol(100)-*OH and **b** Hol(112)-*OH. Yellow and cyan regions represent charge accumulation and depletion, respectively (isosurface: ± 0.005 e/Bohr^3). The iridium and oxygen atoms are depicted as gray and red spheres, respectively, while the IrO_6 octahedra are shaded in gray.

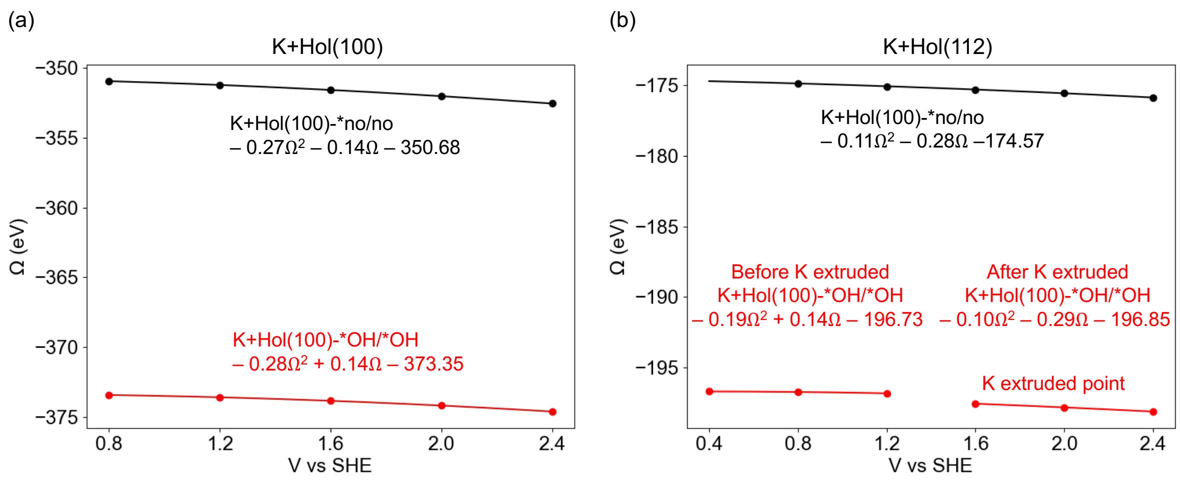

Fig S9. Grand canonical energy as a function of the electrode potential. Example of **a** K+Hol(100) and **b** K+Hol(112).

Table S1. Gibbs energy of each step at 1.6 V for various surfaces in vacuum and grand canonical (GC) conditions. The third column shows the difference ($\Delta G = G_{\mathrm{GC}} - G_{\mathrm{vacuum}}$). All values are in eV. For K+Hol(112), the step sequence differs from the standard path and is highlighted in red. See Fig. S5 for details.

**Surface**	**Step**	**Vacuum**	**GC**	ΔG
Rut(110)	G1	−1.67	−1.71	−0.04
	G2	0.13	0.08	−0.05
	G3	−0.11	−0.45	−0.33
	G5	−0.94	−0.51	0.43
	G6	1.12	1.11	−0.01
Hol(100)	G1	−1.44	−1.48	−0.03
	G2	0.02	−0.01	−0.02
	G3	−0.13	−0.31	−0.19
	G5	−0.64	−0.62	0.02
	G6	0.71	0.94	0.22
Hol(112)	G1	−1.20	−1.23	−0.03
	G2	0.02	0.00	−0.02
	G3	−0.28	−0.32	−0.04
	G5	−0.43	−0.39	0.05
	G6	0.41	0.45	0.05
K+Hol(100)	G1	−1.43	−1.50	−0.07
	G2	0.05	0.05	0.01
	G3	−0.34	−0.51	−0.17
	G5	−0.63	−0.41	0.21
	G6	0.86	0.88	0.02
K+Hol(112)	G1	−1.63	−1.83	−0.20
	G2	−0.20	−0.18	0.02
	G3	−1.05	0.31	1.36
	G5	0.09	−0.72	−0.81
	G6	1.32	0.94	−0.37

Table S2. Bonding contributions between Ir and O in *OH orbitals on various surfaces. −IpCOHP values of each orbital bonding are shown.

Bonding type	Rut(110)	Hol(100)	Hol(112)
Total	4.06	3.97	4.01
$6s$–$2s$ (σ)	0.80	0.77	0.72
$6s$–$2p_y$	0.00	0.00	0.01
$6s$–$2p_z$ (σ)	0.38	0.39	0.37
$6s$–$2p_x$	0.00	0.00	0.00
$5d_{xy}$–$2s$	0.00	0.00	0.00
$5d_{xy}$–$2p_y$	0.00	0.00	0.00
$5d_{xy}$–$2p_z$	0.00	0.00	0.00
$5d_{xy}$–$2p_x$	0.00	0.00	0.00
$5d_{yz}$–$2s$	0.00	0.00	0.00
$5d_{yz}$–$2p_y$ (π)	0.06	0.06	0.22
$5d_{yz}$–$2p_z$	0.00	0.01	0.00
$5d_{yz}$–$2p_x$	0.00	0.00	0.01
$5d_{z^2}$–$2s$ (σ)	0.54	0.52	0.52
$5d_{z^2}$–$2p_y$	0.01	0.00	0.00
$5d_{z^2}$–$2p_z$ (σ)	1.80	1.76	1.85
$5d_{z^2}$–$2p_x$	0.00	0.00	0.00
$5d_{xz}$–$2s$	0.00	0.00	0.00
$5d_{xz}$–$2p_y$	0.00	0.00	0.01
$5d_{xz}$–$2p_z$	0.00	0.00	0.00
$5d_{xz}$–$2p_x$ (π)	0.48	0.46	0.31
$5d_{x^2y^2}$–$2s$	0.00	0.00	0.00
$5d_{x^2y^2}$–$2p_y$	0.00	0.00	0.00
$5d_{x^2y^2}$–$2p_z$	0.00	0.00	0.00
$5d_{x^2y^2}$–$2p_x$	0.00	0.00	0.00

Table S3. Bonding contributions between Ir and O in *O orbitals on various surfaces. −IpCOHP values of each orbital bonding are shown.

Bonding type	Rut(110)	Hol(100)	Hol(112)
Total	6.73	6.79	6.74
$6s$–$2s$ (σ)	1.42	1.43	1.35
$6s$–$2p_y$	0.00	0.00	0.00
$6s$–$2p_z$ (σ)	0.24	0.23	0.23
$6s$–$2p_x$	0.00	0.00	0.00
$5d_{xy}$–$2s$	0.00	0.00	0.00
$5d_{xy}$–$2p_y$	0.00	0.00	0.00
$5d_{xy}$–$2p_z$	0.00	0.00	0.00
$5d_{xy}$–$2p_x$	0.00	0.00	0.00
$5d_{yz}$–$2s$	0.00	0.00	0.00
$5d_{yz}$–$2p_y$ (π)	0.85	0.89	0.98
$5d_{yz}$–$2p_z$	0.00	0.00	0.00
$5d_{yz}$–$2p_x$	0.00	0.00	0.00
$5d_{z^2}$–$2s$ (σ)	0.81	0.78	0.77
$5d_{z^2}$–$2p_y$	0.00	0.00	0.00
$5d_{z^2}$–$2p_z$ (σ)	2.41	2.44	2.54
$5d_{z^2}$–$2p_x$	0.00	0.00	0.00
$5d_{xz}$–$2s$	0.00	0.00	0.00
$5d_{xz}$–$2p_y$	0.00	0.00	0.00
$5d_{xz}$–$2p_z$	0.00	0.00	0.00
$5d_{xz}$–$2p_x$ (π)	1.00	1.01	0.88
$5d_{x^2y^2}$–$2s$	0.00	0.00	0.00
$5d_{x^2y^2}$–$2p_y$	0.00	0.00	0.00
$5d_{x^2y^2}$–$2p_z$	0.00	0.00	0.00
$5d_{x^2y^2}$–$2p_x$	0.00	0.00	0.00

Table S4. Bonding contributions between Ir and O in *OOH orbitals on various surfaces. −IpCOHP values of each orbital bonding are shown.

Bonding type	Rut(110)	Hol(100)	Hol(112)
Total	4.43	4.25	4.21
$6s$–$2s$ (σ)	0.96	0.94	0.95
$6s$–$2p_y$	0.00	0.00	0.00
$6s$–$2p_z$ (σ)	0.36	0.34	0.31
$6s$–$2p_x$	0.00	0.01	0.01
$5d_{xy}$–$2s$	0.00	0.00	0.00
$5d_{xy}$–$2p_y$	0.00	0.00	0.00
$5d_{xy}$–$2p_z$	0.00	0.00	0.00
$5d_{xy}$–$2p_x$	0.00	0.00	0.00
$5d_{yz}$–$2s$	0.00	0.00	0.00
$5d_{yz}$–$2p_y$ (π)	0.32	0.50	0.38
$5d_{yz}$–$2p_z$	0.00	0.00	0.00
$5d_{yz}$–$2p_x$	0.00	0.00	0.00
$5d_{z^2}$–$2s$ (σ)	0.58	0.56	0.55
$5d_{z^2}$–$2p_y$	0.00	0.00	0.00
$5d_{z^2}$–$2p_z$ (σ)	1.69	1.67	1.69
$5d_{z^2}$–$2p_x$	0.00	0.00	0.00
$5d_{xz}$–$2s$	0.00	0.00	0.00
$5d_{xz}$–$2p_y$	0.00	0.00	0.00
$5d_{xz}$–$2p_z$	0.00	0.00	0.00
$5d_{xz}$–$2p_x$ (π)	0.51	0.25	0.34
$5d_{x^2y^2}$–$2s$	0.00	0.00	0.00
$5d_{x^2y^2}$–$2p_y$	0.00	0.00	0.00
$5d_{x^2y^2}$–$2p_z$	0.00	0.00	0.00
$5d_{x^2y^2}$–$2p_x$	0.00	0.00	0.00

Table S5. Integrated Crystal Orbital Hamilton Population (−ICOHP) values for oxygenated intermediates (*OH, *OOH, *O_2, and *O) under vacuum conditions.

**Surface**	***OH**	***OOH**	***O_2**	***O**
Rut(110)	4.13	4.38	4.53	6.86
Hol(100)	4.00	4.23	4.24	6.90
Hol(112)	4.04	4.20	4.11	6.82

Table S6. Integrated Crystal Orbital Hamilton Population (−ICOHP) values for oxygenated intermediates (*OH, *OOH, *O_2, and *O) under grand canonical (GC) conditions.

**Surface**	***OH**	***OOH**	***O_2**	***O**
Rut(110)	4.06	4.56	4.68	6.73
Hol(100)	3.97	4.40	4.41	6.79
Hol(112)	4.01	4.37	4.19	6.74

Table S7. Bader charge of surface Ir atoms for each oxygenated intermediate (*, *OH, *O, *OOH, and *O_2) on various surfaces. All values are in eV.

**Surface**	*****	***OH**	***O**	***OOH**	***O_2**
Rut(110)	+1.98	+2.02	+2.02	+2.01	+2.01
Hol(100)	+1.93	+1.84	+1.91	+1.95	+2.00
Hol(112)	+2.19	+2.15	+2.24	+2.08	+2.14
K+Hol(100)	+1.82	+1.77	+1.96	+1.77	+1.85
K+Hol(112)	+2.04	+2.08	+2.14	+2.11	+1.96

[1] G. Kresse and D. Joubert, From ultrasoft pseudopotentials to the projector augmented-wave method, Phys. Rev. B **59**, 1758 (1999).

[2] G. Kresse and J. Hafner, *Ab Initio* molecular dynamics for liquid metals, Phys. Rev. B **47**, 558 (1993).

[3] G. Kresse and J. Furthmüller, Efficient iterative schemes for *Ab Initio* total-energy calculations using a plane-wave basis set, Phys. Rev. B **54**, 11169 (1996).

[4] J. P. Perdew, K. Burke, and M. Ernzerhof, Generalized gradient approximation made simple, Phys. Rev. Lett. **77**, 3865 (1996).

[5] S. Grimme, S. Ehrlich, and L. Goerigk, Effect of the damping function in dispersion corrected density functional theory, J. Comput. Chem. **32**, 1456 (2011).

[6] K. Momma and F. Izumi, VESTA: a three-dimensional visualization system for electronic and structural analysis, J. Appl. Crystallogr. **41**, 653 (2008).

[7] S. M. Islam, F. Khezeli, S. Ringe, and C. Plaisance, An implicit electrolyte model for plane wave density functional theory exhibiting nonlinear response and a nonlocal cavity definition, J. Chem. Phys. **159**, 234117 (2023).

[8] S. Trasatti, Interfacial behaviour of non-aqueous solvents, Electrochim. Acta **32**, 843 (1987).

[9] T. Cheng, L. Wang, B. V. Merinov, and W. A. I. Goddard, Explanation of dramatic ph-dependence of hydrogen binding on noble metal electrode: Greatly weakened water adsorption at high ph, J. Am. Chem. Soc. **140**, 7787 (2018).

[10] G. Henkelman, B. P. Uberuaga, and H. Jónsson, A climbing image nudged elastic band method for finding saddle points and minimum energy paths, J. Chem. Phys. **113**, 9901 (2000).

[11] R. Dronskowski and P. E. Blöchl, Crystal orbital hamilton populations (cohp): Energy-resolved visualization of chemical bonding in solids based on density-functional calculations, J. Phys. Chem. **97**, 8617 (1993).

[12] V. L. Deringer, A. L. Tchougréeff, and R. Dronskowski, Crystal orbital hamilton population (cohp) analysis as projected from plane-wave basis sets, J. Phys. Chem. A **115**, 5461 (2011).

[13] S. Maintz, V. L. Deringer, A. L. Tchougréeff, and R. Dronskowski, Analytic projection from plane-wave and paw wavefunctions and application to chemical-bonding analysis in solids, J. Comput. Chem. **34**, 2557 (2013).

[14] S. Maintz, V. L. Deringer, A. L. Tchougréeff, and R. Dronskowski, Lobster: A tool to extract chemical bonding from plane-wave based dft, J. Comput. Chem. **37**, 1030 (2016).

[15] S. Maintz, M. Esser, and R. Dronskowski, Efficient rotation of local basis functions using real spherical harmonics, Acta Phys. Pol. B **47**, 1165 (2016).

[16] R. Nelson, C. Ertural, J. George, V. L. Deringer, G. Hautier, and R. Dronskowski, Lobster: Local orbital projections, atomic charges, and chemical-bonding analysis from projector-augmented-wave-based density-functional theory, J. Comput. Chem. **41**, 1931 (2020).

[17] M. D. Hossain, Y. Huang, T. H. Yu, W. A. I. Goddard, and Z. Luo, Reaction mechanism and kinetics for co_2 reduction on nickel single atom catalysts from quantum mechanics, Nat. Commun. **11**, 2256 (2020).

[18] J. K. Nørskov, J. Rossmeisl, A. Logadottir, L. R. K. J. Lindqvist, J. R. Kitchin, T. Bligaard, and H. Jonsson, Origin of the overpotential for oxygen reduction at a fuel-cell cathode, J. Phys. Chem. B **108**, 17886 (2004).

[19] I. C. Man, H.-Y. Su, F. Calle-Vallejo, H. A. Hansen, J. I. Martínez, N. G. Inoglu, J. Kitchin, T. F. Jaramillo, J. K. Nørskov, and J. Rossmeisl, Universality in oxygen evolution electrocatalysis on oxide surfaces, ChemCatChem **3**, 1159 (2011).
